# *Mucilaginibacter* sp. K Improves Growth and Induces Salt Tolerance in Nonhost Plants *via* Multilevel Mechanisms

**DOI:** 10.3389/fpls.2022.938697

**Published:** 2022-06-27

**Authors:** Di Fan, Donald L. Smith

**Affiliations:** ^1^School of Biology, Food and Environment, Hefei University, Hefei, China; ^2^Department of Plant Science, McGill University, Montreal, QC, Canada

**Keywords:** *Mucilaginibacter* sp., plant growth promotion, salt stress, *Zea mays* L., Arabidopsis

## Abstract

Soil salinity negatively modulates plant growth and development, contributing to severe decreases in the growth and production of crops. *Mucilaginibacter* sp. K is a root endophytic bacterium that was previously reported by our laboratory to stimulate growth and confer salt tolerance in Arabidopsis (*Arabidopsis thaliana*). The main purpose of the present study is to elucidate the physiological and molecular machinery responsible for the prospective salt tolerance as imparted by *Mucilaginibacter* sp. K. We first report that auxin, gibberellin, and MPK6 signalings were required for strain K-induced growth promotion and salt tolerance in Arabidopsis. Then, this strain was assessed as a remediation strategy to improve maize performance under salinity stress. Under normal growth conditions, the seed vigor index, nitrogen content, and plant growth were significantly improved in maize. After NaCl exposure, strain K significantly promoted the growth of maize seedlings, ameliorated decline in chlorophyll content and reduced accretion of MDA and ROS compared with the control. The possible mechanisms involved in salt resistance in maize could be the improved activities of SOD and POD (antioxidative system) and SPS (sucrose biosynthesis), upregulated content of total soluble sugar and ABA, and reduced Na^+^ accumulation. These physiological changes were then confirmed by induced gene expression for ion transportation, photosynthesis, ABA biosynthesis, and carbon metabolism. In summary, these results suggest that strain K promotes plant growth through increases in photosynthesis and auxin- and MPK6-dependent pathways; it also bestows salt resistance on plants through protection against oxidative toxicity, Na^+^ imbalance, and osmotic stress, along with the activation of auxin-, gibberellin-, and MPK6-dependent signaling pathways. This is the first detailed report of maize growth promotion by a *Mucilaginibacter* sp. strain from wild plant. This strain could be used as a favorable biofertilizer and a salinity stress alleviator for maize, with further ascertainment as to its reliability of performance under field conditions and in the presence of salt stress.

## Introduction

Due to global population expansion, decreases in arable land and developing climate change conditions, we are facing an impending food crisis. Crop production is fundamentally impacted by plant growth and development and how they cope with abiotic and biotic stresses. Climate change and its associated impacts on the environment, such as high salt concentrations, increased precipitation, warming, and water deficit, could well diminish the current level of global food security (Gojon et al., 2022).

Soil salinization is one of the major abiotic pressures that compromise crop growth and productivity worldwide, mainly due to osmotic stress, reactive oxygen species (ROS) accumulation, and ionic toxicity (Ilangumaran and Smith, 2017). Salinity induces negative effects on plant metabolism; excessive levels of salt cause abnormal growth and development eventually resulting in low yield. In the course of evolution, plants have developed ubiquitous mechanisms responses to salt tolerance, such as increasing anti-oxidative systems, buildup of compatible compounds, ion balancing, and compartmentation of toxic ions (Zhu, 2016). These specific strategies lead to the activation of signaling pathways to combat salinity stress by maintaining cellular homeostasis (Aslam et al., 2021). Phytohormones, such as auxin, ethylene, and abscisic acid (ABA), are known to be actively involved in coping with salt stress, through adjustment of whole-plant metabolism (Khan et al., 2020).

Salinity resistance can be enhanced by genetic manipulation of crop plants, leading to both agronomic and economic benefits; however, there has been debate regarding health and ecological risks associated with modified genes (Zhang et al., 2016), limiting their use for intensive agricultural production. In this case, “Organic Agriculture” is a production system that minimizes the use of synthetic chemicals in non-genomic–modified plants and promotes soil productivity, based on sustainable agricultural practices such as crop rotation, biocontrol, and biofertilization. Endogenous applications of plant bio-stimulants (BSs; microorganisms or natural substances) have recently aroused enormous attention globally. As potentially novel, environmentally friendly, cost-effective, and sustainable approaches, aside from growth promotion, they can further improve abiotic stress resistance in plants (Drobek et al., 2019; Sani and Yong, 2021). Numerous plant growth-promoting rhizobacteria (PGPR), which belong to bacterial-based BSs, have been adopted as sustainable agronomic practices leading to improved plant growth and yield and reduced susceptibility to biotic and/or abiotic stresses in crop plants, such as soybean, wheat, and pepper (Hahm et al., 2017; Khande et al., 2017; Vries et al., 2020).

Many PGPRs promote plant growth and mitigate salt stress through effects on carbon and energy metabolism, ion selectivity, nutrient transportation, defense systems, and phytohormone biosynthesis in plant (Tabassum et al., 2017; Alkahtani et al., 2020). For example, *Alcaligenes faecalis* JBCS1294 has been reported to confer salinity tolerance in Arabidopsis through the auxin and gibberellin pathways and upregulation of ion transport genes in the roots (Bhattacharyya et al., 2014). Arabidopsis treated with multi-traits rhizobacterial strains, *Pseudomonas*, *Bacillus*, and *Rhizobium* spp., respectively, showed enhanced salinity tolerance by regulating osmolytes and antioxidant systems (Fan et al., 2020). A halotolerant PGPR, *Bacillus* sp. SR-2-1/1, augmented salt tolerance in maize through attenuating oxidative damage, enhancing antioxidative status, and maintaining ionic homeostasis (Rafiq et al., 2020). More recently, the work from Andres-Barrao et al. showed that the coordinated regulation of the sulfur metabolic pathways in both *Enterobacter* sp. SA187 and Arabidopsis conferred salinity stress tolerance to host plants (Andres-Barrao et al., 2021). *Streptomyces albidoflavus* OsiLf-2 inoculation onto rice plants in hydroponic and saline-alkaline soil conditions (growth-chamber and field) increased plant growth and stress tolerance by increasing osmolytes and photosynthesis efficiency, as well as enhancing transcriptional levels of genes related to stress-response and ion-transport (Niu et al., 2021). Regardless of the alleviation ability of PGPRs under salinity, the interactions between plants and microbes are numerous and complex, and the underlying mechanisms are worth further delineating.

Maize (*Z. mays* L.) is the third most important cereal crop, providing high economic returns but accompanied by high production costs stem from tillage, irrigation, fertilizer, and pesticides. Besides its preeminence as food and livestock feed, maize has emerged as a biofuel crop, which provides renewable and cleaner-burning alternatives to fossil fuels (Ma et al., 2017), leading to a 46% reduction in greenhouse gas emission compared to regular gasoline.^1^ The production of maize can be severely constrained by environmental stresses, including soil salinity (Farooq et al., 2015). In this situation, growing maize in less fertile and/or salt affected soils may become successful if a relatively prompt growth and high economic yield could be achieved with relatively small input costs.

Our earlier experiments showed that a *Mucilaginibacter* sp. K from wild perennial herbaceous plant *Scorzoneroides autumnalis* elicited systemic physiological responses and modulated a wide set of genes involved in stress response and hormone metabolism, leading to improved growth and salt tolerance in *Arabidopsis thaliana* (L.) Heynh. Columbia-0 (Fan et al., 2020). *Mucilaginibacter*, belonging to the family Sphingobacteriaceae, is known to hydrolyze organic matter, such as xylan and pectin, and produce enormous amounts of extracellular polymeric substances (EPS; Han et al., 2012). Though possessing various PGP traits, to date, *Mucilaginibacter* spp. have been explored in only three studies regarding their growth promotion effect on plants, specifically, Arabidopsis and maize (Fan et al., 2020; Fan and Smith, 2021), cannabis (Lyu et al., 2022), and canola and tomato (Madhaiyan et al., 2010). However, no systematic research on their effects in major crops, such as maize, have been reported. With this in mind, the present work was formulated to evaluate the effects of *Mucilaginibacter* sp. K on maize subjected to NaCl-induced salinity stress on the basis of physiological, biochemical, and molecular changes. To the best of our knowledge, the present is also the first attempt to decipher the signaling-responsive pathways in Arabidopsis interfered with a *Mucilaginibacter* sp. upon exposure to salinity.

## Materials and Methods

### Bacterial Strain

*Mucilaginibacter* sp. K was selected for its potential as a biofertilizer and stress (salt) alleviator (Fan et al., 2020). It was originally isolated from roots of fall dandelion in Sainte-Anne-de-Bellevue, Québec, Canada (Fan et al., 2018). This strain was maintained on King’s B medium (KB) at 28°C for 3 days. The standardized bacterial suspension was prepared in 10 mM MgSO_4_ as described (Fan et al., 2020) and was used for bacterization experiments.

### Plant Materials

Wild-type *A. thaliana* (L.; Columbia), *Arabidopsis* salicylic acid-deficient transgenic *NahG* line (Munné-Bosch et al., 2007), as well as the signaling mutated lines *mpk3-1* (SALK_151594; Wang et al., 2007a), *mpk6-2* (SALK_073907; Liu and Zhang, 2004), *eir1-1* (CS8058; Roman et al., 1995), *jar1-1* (CS8072; Staswick et al., 2002), *npr1-5* (CS3724; Zipfel et al., 2004), *etr1-3* (CS3070; Zipfel et al., 2004), and *gai* (SAIL_587_C02; Hu et al., 2021; all in a Col-0 background, except for *npr1-5* in a No-0 background) were obtained from Dr. Fangwen Bai (Institut de Recherche en Biologie Végétale, Université de Montréal). Seeds were treated with bacterial suspensions and grown in petri dishes as previously described (Fan et al., 2020). Hydroponics growth of Arabidopsis was done as previously described (Conn et al., 2013). In short, 21-day-old seedlings were transferred to an aerated tank containing modified 1/4 Hoagland’s solution supplemented with 230 mM NaCl and observed every 6 h until the seedlings were completely withered in the control.

Non-treated hybrid maize seeds (var. 19K19) were surface sterilized with 70% ethanol (Marques et al., 2010). The seeds were then air dried in a laminar flow hood, and were sown in pots or treated at room temperature for 1 h in the absence of light with dense (10^9^ cfu ml^−1^) suspensions of the bacterial strains in sterile 10 mM MgSO_4_ or sterile 10 mM MgSO_4_ (as a negative control), while a small subset of the seeds was placed onto Difco™ Nutrient broth (NB) agar plates and incubated at 28°C for 7 d, to check for any microbial contamination.

### Determination of MPK Phosphorylation

The 14-day-old Col-0 seedlings were pre-inoculated with strain K or not were left untreated or treated with 100 mM NaCl for the indicated periods of time. Arabidopsis leaves were ground in liquid nitrogen and then homogenized in extraction buffer [25 mM Tris–HCl (pH 7.5), 150 mM NaCl, 1 mM EDTA, 0.5% Triton-X100, 25 mM NaF, 25 mM Na_3_VO_4_, 5 mM dithiothreitol (DTT), 20% glycerol, and complete Protease Inhibitor Cocktail], after which the slurry was centrifuged at 13,000 × *g* for 20 min at 4°C. MPK activity was determined by Western blot (Jiang et al., 2016) using anti-p-ERK [anti-pERK MAPK (Phospho-p44/p42 MAPK, ERK1/2, Thr202/Tyr204, D13.14.4E), Cell Signaling Technology] that specifically recognizes the [T-X-Y] MPK activation motif as the primary antibody. A total of 30 μg aliquots of total protein from each sample were fractionated on a 10% SDS-PAGE and subsequently blotted onto a nitrocellulose membrane (Millipore 0.2 μM). The blot was then stained with 0.1% (w/v) ponceau-S staining solution to visualize total protein and to verify equal loading of total protein for each sample. The secondary antibody was HRP conjugated Goat anti-Rabbit IgG(H + L; Thermo Fisher Scientific).

### Assay for Inhibition of Signaling Molecules

A total of 5 μM of aminoethoxyvinylglycine (AVG), 20 μM of daminozide (DZ), and 10 μM of silver nitrate (SN) were used as auxin, gibberellin, and ethylene biosynthesis inhibitors, respectively. Arabidopsis Col-0 seeds were treated with strain K and sown on half-strength MS media supplemented with signaling inhibitors and 100 mM NaCl. The growth performance was investigated for up to 14 days of incubation.

### *In vitro* Bacterial Impact on Salinity Tolerance of Maize

*In vitro* inoculation of maize with *Mucilaginibacter* sp. K was conducted as previously described (Fan and Smith, 2021), and 21-day-old seedlings were harvested for physiological and biochemical measurements as well as RT-qPCR analysis. Salt stress treatment (100 mM NaCl) was initiated after 10 days of growth in glass tubes. Salt injury index (SI) analysis (Sivritepe et al., 2008) was conducted based on a 1–4 scale of visual quality of maize leaves as follows: 1, no injury; 2, browning on shoot-tips and leaf edges; 3, necroses on the whole leaf and/or on part of the stem; and 4, dead. SI was then calculated using the following formula: SI = ∑ (ni × i)/*N*, where ni is the number of seedlings receiving the assessment “i” (from 1 to 4), and *N* is the total number of seedlings in each treatment.

### Biochemical Measurements

#### Determination of Lipid Peroxidation

The malondialdehyde (MDA) concentration was measured using the thiobarbituric acid (TBA) method (Hodges et al., 1999). Fresh leaf samples were homogenized in 80% EtOH. The supernatant and distilled water were added to a test tube with either (i) –TBA (thiobarbituric acid) solution containing 20% (w/v) trichloroacetic acid (TCA) and 0.01% (w/v) butylated hydroxytoluene (BHT), or (ii) +TBA solution containing the above plus 0.65% (w/v) TBA. The mixture as then mixed vigorously heated at 95°C in a dry bath, cooled, and centrifuged. Absorbances were measured at 440, 532, and 600 nm. MDA equivalents were calculated using the following formula: (1) [(Abs 532 + TBA) – (Abs 600 + TBA) – (Abs 532-TBA-Abs 600 – TBA)] = A, (2) [(Abs 440 + TBA) – (Abs 600 + TBA) 0.0571] = B, (3) MDA equivalents (nmol ml^−1^) = 106 [(A – B) / 157,000].

#### Determination of the Content of Total Proteins, Total Soluble Sugars, and Total Proline

Proline estimation was performed following previously described procedures (Fan et al., 2020), using an acid ninhydrin method. Chlorophyll content was quantified according to Fan et al. (2020), except maize leaves were ground in combination with quartz sand.

For TSS (total soluble sugar estimation; Singh and Jha, 2016), 50 mg maize leaf tissue were homogenized in 3 ml 80% ethanol, followed by centrifugation at 10,000 × *g* at 4°C for 5 min to remove insoluble material. To 100 μl of sample was added 3 ml of freshly prepared anthrone reagent, and the mixture was allowed to react in a boiling water bath. After cooling on ice, absorbance was read at 620 nm against 80% ethanol as a blank. TSS was expressed as glucose equivalent by comparison with a glucose standard curve. The antiradical activity of maize leaves was measured *via* a DPPH (2,2-diphenyl-1-picrylhydrazyl hydrate) assay (Fan et al., 2020).

The extraction of total soluble protein was conducted as described (Fan et al., 2011). Leaf tissues were extracted in 0.1 M potassium phosphate buffer (pH 8.0), containing 5.0 mM β-mercaptoethanol and 2% (w/v) PVP, and centrifuged at 10,000 × *g* for 20 min at 4°C. The supernatant was collected as the crude enzyme. Protein concentration was determined with Bradford’s reagent, with bovine serum albumin as a reference standard.

#### Determination of Reactive Species

Superoxide (•O_2_^−^) content was estimated as described (Liu et al., 2010). Fresh maize leaf tissue was homogenized in liquid nitrogen with 65 mM potassium phosphate buffer (pH 7.8) followed by centrifugation at 5,000 × *g* for 10 min. The reaction mixture composed of 1 ml of the supernatant, 0.9 ml of 65 mM phosphate buffer (pH 7.8), and 0.1 ml of 10 mM hydroxylamine hydrochloride was incubated, after which 1 ml of 17 mM sulfanilamide and 7 mM α-naphthylamine, respectively, were added for another round of incubation. The absorbance was read at 530 nm and the production rate of •O_2_^−^ was calculated against a standard curve prepared with NaNO_2_.

Hydrogen peroxide (H_2_O_2_) content in maize was estimated as previously described (Velikova et al., 2000). Briefly, fresh leaf tissue was extracted with trichloro acetic acid (TCA). The supernatant was mixed with 10 mM potassium phosphate buffer (pH 7.0) and 1 M potassium iodide (KI), after which the absorbance was read at 390 nm.

#### Enzyme Activities Assays

Crude enzyme was extracted from deveined maize leaves in 50 mM potassium phosphate buffer (pH 7.5) at 4°C. Activities of catalase (CAT, EC1.11.1.6) and peroxidase (POD, EC1.11.1.7) were assessed spectrophotometrically as previously described (Fan et al., 2020), based on the decomposition of H_2_O_2_ and formation of purpurogallin, respectively. Superoxide dismutase (SOD, EC1.15.1.1) activity was monitored by the production of sucrose from uridin 5′-diphosphate (UDP)-glucose (Rufty and Huber, 1983; Saher et al., 2005). The activity of sucrose phosphate synthase (SPS, EC2.4.1.14) was examined following a NBT (nitroblue tetrazolium) photoreduction method (Abedi and Pakniyat, 2010).

### Seed Germination Assay

Seedling vigor was assayed to test strain K for its plant growth promoting ability, specifically, with regard to the effects on seed germination, root and hypocotyl growth (Abdul-Baki and Anderson, 1973). Inoculated maize seeds were arranged in an equidistant manner in a Petri dish previously lined on the bottom with two layers of Fisherbrand P8 filter paper moistened with 10 ml of sterile ddH_2_O. The plates were sealed with Parafilm and incubated at a constant temperature of 25°C in the dark inside a growth chamber. Seed germination was evaluated daily for up to 7 days. A seed was considered germinated when its radicle was at least 1 mm in length. Germination percentage (GP) was computed on the day 7 by the following formula: GP = (total number of normal germinating seeds/total number of experimental seeds, in all replicates) × 100. Seedling vigor characteristics (lengths of roots and hypocotyls), as well as seedling fresh and dry weight were recorded. The vigor index (VI) was calculated using the following formula: VI = % germination × (mean root length plus mean hypocotyl length). There were 10 replications each with 10 bacterially treated seeds, or in the case of the control, 10 untreated seeds. The experiment was conducted twice (repeated).

### Evaluation of Root Colonization

Maize root colonization potential of strain K was determined *via* serial dilution plating, using seedlings from the germination assay. Randomly chosen seedlings of maize were rinsed in sterile ddH_2_O five times and then patted dry with sterile paper towels. The root samples were surface sterilized with 70% ethanol for 10 min, rinsed three times with sterile ddH_2_O, and then shaken for 5 min in commercial bleach (3% available chlorine), followed by 5 changes of sterile ddH_2_O. To confirm tissue surface disinfection, a 100 μl sample of the sterile ddH_2_O water from the final rinse were spread on KB plates and bacterial growth was assessed following a 7-day incubation period at 28°C. Following sterilization, the root samples were crushed in sterilized PBS and the supernatants were serially diluted, spread on KB agar plates supplemented with kanamycin, and incubated at 28°C. After 1–4 days, number of viable cells was estimated as colony forming units (CFUs) g^−1^ root (FW). This experiment was repeated three times. Identity of the isolated strains were confirmed by 16S rDNA sequencing (Fan et al., 2020).

### Transcription Analysis

To elucidate the role of strain K in improving maize growth under saline conditions, real-time expression of genes involved in carbohydrate metabolism and ion transportation were performed. Total RNA extraction, cDNA synthesis, and gene expression analysis were conducted as previously described (Fan and Smith, 2021). Gene-specific primers were designed and verified using Primer-Blast (NCBI). The primers used for qRT-PCR are listed in Supplementary Table S1. The relative transcript levels of the analyzed genes were normalized to *Ubi2* as an internal reference control. Maize seedlings grown without strain K inoculation served as control group. The relative-fold changes were calculated using the 2^–∆∆CT^ method (Livak and Schmittgen, 2001).

### Extraction and Quantitative Measurement of ABA

Fresh maize leaves were processed as described (Arkhipova et al., 2007), with modifications. Plant tissues were homogenized in 80% ethanol and incubated overnight at 4°C followed by centrifugation, after which the supernatant was collected. ABA content was detected by the enzyme-linked immunosorbent assay (ELISA), using the ABA ELISA kit (MBS 282218, 48 tests, MyBioSource Inc.).

### Impact of Strain K on Greenhouse Growth of Maize Plants

#### Plant Growth

To evaluate the efficiency of *Mucilaginibacter* sp. K on the growth variables of maize, a pot experiment was conducted. The potting mixture (1 Turface: 2 sand, v/v) was weighed (1,800 × *g*) for each pot (top diameter of 150 mm), after which the pot was moistened with Hoagland’s nutrient solution, diluted to 25% in ddH_2_O, the day before sowing.

Each pot received four sterilized maize seeds of equivalent size and shape, sown at a depth of 2.5 cm. The seeds were then immediately inoculated with 10 ml of bacterial suspension. Pots were organized following a completely randomized design and maintained under greenhouse conditions with average day and night temperatures of 26°C and 18°C, respectively, and natural light. After 7 days of sowing (DAS), seedlings were thinned to one per pot and received a second inoculum dose at 10 ml per plant. Uninoculated control plants were mock-inoculated with 10 mM MgSO_4_. During the growth period, plants were given equal amounts of ½ Hoagland’s solution twice a week.

#### Growth Variable and Pigment Determination

Chlorophyll content index of maize leaves was recorded using a portable, handheld chlorophyll meter (SPAD-502, Konica Minolta), measured as the optical density. In total, four fully expanded leaves, from each pot of eight plants per treatment, were used for measurement and two readings per leaf (one on each side of the main vein) were recorded. Photosynthetic rate was taken on fully expanded leaves using a LI-6400 portable system (LI-COR®). All plants were well irrigated before this measurement was taken. The data on measured growth variables were collected from 35 DAS.

After the vegetative growth was harvested, the height and circumference were measured. For root length measurement, samples were washed in ddH_2_O to removed debris, after which an 8-bit grey scale image of each root sample was acquired by digital scanning at a 400 dots per inch (dpi) resolution using a flatbed image scanner (Modified Epson Expression 10000XL; Epson America, Inc., San Jose, CA, United States), in which the root lengths were measured using commercial WinRHIZO software (Regent Instruments Inc., Montreal, Québec, Canada). Total leaf area was measured with a LI-3100C Area Meter (LI-COR®), according to manufacturer’s instructions. The above-ground and root biomass were collected and weighed after drying in an oven at 85°C for 72 h.

#### Antioxidative Activities

The total antioxidant capacity of maize leaves was determined using a DPPH (2,2-diphenyl-1-picrylhydrazyl hydrate) assay as described by Awika et al. (2003). Dried maize leaf tissue was homogenized and extracted in pure methanol (MeOH). The supernatant was added to fresh DPPH solution (0.11 mM) and incubated for 1 h at 22°C in the dark. The absorbance of the samples was then read at 515 nm. The results were expressed in mg Trolox equivalents (TE, mg Trolox)/100 g DW through comparison against a Trolox standard curve (25–800 μM), which was conducted in parallel. Total phenolic compounds were analyzed as described by Fan et al. (2011), with slight modifications. Fresh maize leaves (0.5 g) were frozen in liquid N_2_ and extracted twice with 70% MeOH, after which the supernatant was mixed with 2 N Folin–Ciocalteu reagent. Total phenolics were expressed as gallic acid equivalents (GAE, mg gallic acid g^−1^ FW).

#### Estimation of Water-Soluble Carbohydrate

Total soluble carbohydrate was estimated using the phenol-sulfuric acid method (DuBois et al., 1956). Briefly, 50 mg of the dried leaf tissue was weighed into a glass tube with 2.5 ml of 2.5 N HCl and kept in a boiling water bath for 3 h. After neutralization with solid sodium carbonate, the volume was made up to 50 ml and centrifuged at 8,000 × *g* for 10 min, to remove insoluble material. To 100 μl of sample was added, consecutively, 100 μl of 5% phenol water solution, and 500 μl of concentrated sulfuric acid. After 10 min of shaking at 100 rpm, the tubes were placed in a water bath at 25°C for 20 min. The absorbance of the green colored product, formed by hydroxymethyl furfural reaction with phenol, was read at 490 nm against water as a blank. The total soluble sugar was expressed as glucose equivalent by comparison with a glucose standard curve (0–20 mg).

#### Determination of Nutrient Uptake

Maize plants were washed three times with distilled water. All the shoots or roots were dried in an oven (85°C for 72 h) to obtain a constant weight. They were then combined and ground to a fine powder in a handheld electric coffee grinder. The concentrations of minerals, namely, sodium (Na), potassium (K), calcium (Ca), magnesium (Mg), iron (Fe), zinc (Zn), copper (Cu), and phosphorous (P), were determined using ICP-OES (Nassar et al., 2012) by Kebs Technologies Inc. (Laval, Canada). Samples were incubated overnight in a fume hood in 3.0 ml of nitric acid (HNO_3_) in a 10 ml Oak Ridge centrifuge tube (Thermo Scientific, NY, United States). The following day, samples were digested using a heating block (Thermolyne heater type 16,500 dri-bath model DB16525; Thermolyne, Dubuque, IA 52001, United States) at 105°C until no nitrous oxide gases (brown gases) are evolved, allowed to cool, and diluted 4 times with Milli-Q water and injected into the ICP-OES apparatus for analysis. An elemental stock standard solution mixture (SCP Science, Baie-D’urfe, QC, Canada) was used to calibrate the instrument before sample injection. An inductively coupled argon plasma optical emission spectrometer was used for mineral analysis (model VISTA-MPX CCD, ICPOES, Varian, Australia PTY Ltd., Australia). The concentrations of P, Mg, Ca, and K were expressed as mg g^−1^ of sample dry matter, whereas the Fe, Na, and Cu concentrations were expressed as μg g^−1^ of sample dry matter. Total carbon (C) and nitrogen (N) was analyzed with a Shimadzu TOC-V analyzer (Shimadzu Corporation, Kyoto, Japan) and expressed as % (concentration initially in 100 g of dry matter of leaf or root tissue).

### Statistical Analysis

All experiments were repeated for three times unless otherwise stated. Experimental data were subjected to statistical analysis using one-way analysis of variance (ANOVA) and comparisons between treatment means were calculated using Tukeys Honestly Significant Differences (HSD) test of the COSTAT® statistical software. Graphics were drawn with GraphPad Prism version 9.0 (GraphPad Software, Inc., United States).

## Results

### *Mucilaginibacter* sp. K Regulates Salt Tolerance by MPK6

**Figure 1:**
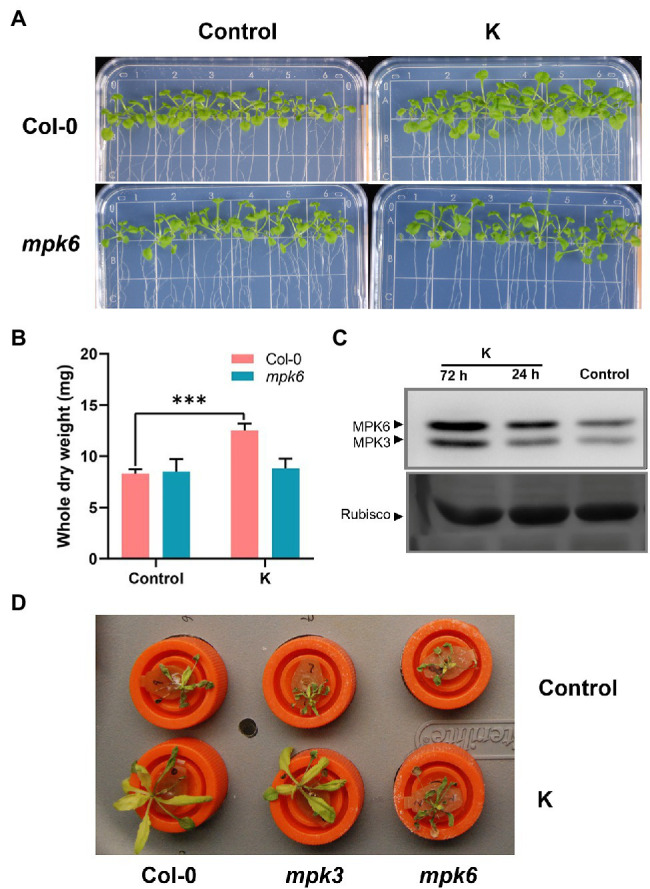
*Mucilaginibacter* sp. K effects on the morphometric traits and MPKs activity of Arabidopsis. Effect of *mpk6* mutant on the phenotype **(A)** and dry weight **(B)** of seed-inoculated Arabidopsis after 21 days of growth. A total of 10 plants were pooled for each replicate for dry weight. Error bars indicate SEM (*n* > 10; ***p < 0.001). **(C)** Activation of MPKs in 14-day-old Arabidopsis Col-0 treated with strain K. Samples were collected at 24 and 72 h after inoculation. Rubisco in ponceau-S stained gel is shown as the loading control. The experiments were performed three times with similar trends. **(D)** Salt resistance by strain K is compromised in *mpk6* mutant. All seedlings were seed-treated with strain K and hydroponically cultured for 21 days. The rosette was completed withered after 18 h exposure to 230 mM NaCl in *mpk6* mutants in either absence or presence of strain K.

While growth stimulation was imposed strain K was still functional in MPK3 mutants (Fan and Smith, 2021), with no discernible difference observed between control and bacterium-treated plants on *mpk6* mutants, indicating strain K-mediated growth promotion was jeopardized in *mpk6* mutant plants (Figures 1A,B). Our previous work showed marginal but significant upregulation of *MPK3* and *MPK6* genes at 12 h post-inoculation of strain K (Fan and Smith, 2021). Here, we monitored whether MPKs were activated in Col-0 after strain K inoculation. As a result, activities of both MPK3 and MPK6 were significantly enhanced in strain K-bioprimed plants. Notably, an increase in the MPK6 activation could be detected in the leaves of K-inoculated seedlings as early as 24 h post-inoculation, and until 3 days post-inoculation the MPKs maintained notable activation (Figure 1C). To test whether MPK3/6 are involved in K-induced salt resistance, the effects of strain K on salinity resistance in *mpk3* and *mpk6* plants were investigated. As a result, Col-0 and *mpk3* Arabidopsis plants but not *mpk6* mutant could be protected by strain K after 18 h exposure to NaCl (Figure 1D). Though MPK3 was also activated by strain K (Figure 1C), taken together, we concluded that MPK6 is required for strain K-mediated salinity resistance, while MPK3 is not critical.

### Effect of *Mucilaginibacter* sp. K on Growth and Salt Tolerance of Arabidopsis Signaling Mutants

**Figure 2:**
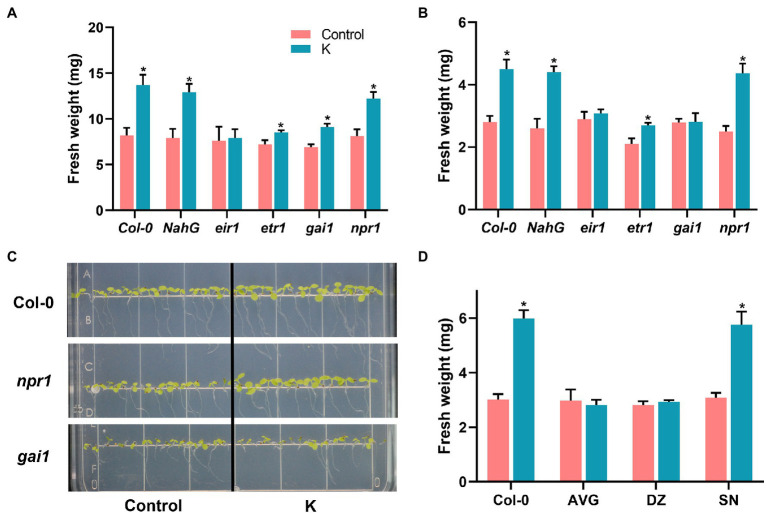
*Mucilaginibacter* sp. K effects on growth performance of Arabidopsis Col-0 and mutant lines under unstressed **(A)** and salt stress **(B)** conditions. Arabidopsis seeds treated with strain K were grown on MS medium supplemented with 100 mM NaCl or not. Fresh weight was recorded 21 days (unstressed) or 14 days (salt stress) after inoculation. **(C)** Representative 7-day-old seedlings of Col-0, *npr1*, and *gai1* grown on salt media were photographed. Inoculated seeds were sown on MS medium supplemented with 100 mM NaCl. **(D)** Effects of strain K on Arabidopsis growth treated with hormone inhibitor under salt stress. Arabidopsis seedlings (4-day-old) were transferred to MS medium supplemented with hormone inhibitors and 100 mM NaCl for 10 days. Data are presented as standard errors of six replicates (*p < 0.05).

We previously found in Arabidopsis that strain K could not prime the whole plant for induction of effective ISR against *Botrytis cinerea* or Pst DC3000 infection (Fan and Smith, 2021). Moreover, strain K led to higher transcription of salicylic acid (SA)-dependent *PR1* gene following salinity stress (Fan and Smith, 2021). To determine the roles of different hormone signaling pathways for growth promotion and salinity tolerance following strain K inoculation, the alterations in seedling fresh weight of Arabidopsis mutant lines were assessed. As indicated in Figure 2A, under normal conditions, strain K significantly increased fresh weight in Col-0, *npr1*, *etr1*, *gai-1*, and NahG plants by 67.1%, 50.6%, 18.1%, 31.9%, and 63.3%, respectively, as compared with control. Conversely, *eir1* mutant line completely lost their ability to interact with strain K, illustrating that auxin signaling is necessary for K-induced growth promotion in Arabidopsis; while salicylate, ethylene, and gibberellin pathways may be independent for growth promotion in response to the inoculation of *Mucilaginibacter* sp. K under unstressed conditions.

We further compared levels of strain K-induced protection against salinity among Arabidopsis Col-0, the defense-signaling mutants, and the transgenic NahG. Pre-inoculation with strain K led to enhanced salinity resistance in Col-0, *npr1*, *etr1*, and NahG plants with fresh weight increased by 60.7%, 74.8%, 28.6%, and 69.2%, respectively, indicating that SA- and ET-dependent signaling pathways are dispensable for K-induced salinity stress (Figure 2B). In contrast, mutants impaired in either gibberellin sensitivity (*gai1*) or auxin transport deficient (*eir1*) showed no significant differences in fresh weight in comparison with control (Figures 2B,C), demonstrating that strain K-induced salinity tolerance relied on auxin- and gibberellin-dependent priming mechanisms.

### Effect of Signaling Inhibitors on Salt Tolerance of Bacterium-Treated Arabidopsis

Based on the results obtained from above, we tested the effects of signaling inhibitors AVG (for auxin), DZ (for gibberellin), and SN (for ethylene) on growth performance of Arabidopsis Col-0 under salinity stress. Again, we failed to observe the elevated fresh weight in AVG and DZ treated seedlings compared to Col-0 grown under salinity stress; while SN treatment led to an 87% increase in fresh weight (Figure 2D). These results further reinforced that auxin and gibberellin signaling are responsible for strain K-mediated salinity tolerance (Figure 2D).

### Effect of *Mucilaginibacter* sp. K on *in vitro* Germination and Growth of Maize

Inoculation of maize seeds with *Mucilaginibacter* sp. K had significant effects on shoot length, whole seedling fresh weight and vigor index (Table 1). When under optimal conditions, strain K caused a 1.28-fold increase in shoot length over the uninoculated control. The fresh weight of seedlings was significantly increased in bio-primed maize seeds (1.25-fold), while the dry weight was unaffected. Seed vigor index was also increased by 16.6% compared to controls.

**Table 1:** Effect of *Mucilaginibacter* sp. K on maize germination parameters.

	Germination (%)	Shoot length (cm)	Root length (cm)	Vigor index	Fresh weight (g)*^1^*	Dry weight (g)*^1^*
Control	83.3	6.4	14.0	1769	0.83	0.28
K	88.3	8.2^***^	13.2	2062^*^	1.04^***^	0.32

* *Indicates differences significant at p < 0.05.*

*** *Indicates differences significant at p < 0.001.*

1 *Values are presented as germinated seed weight.*

**Figure 3:**
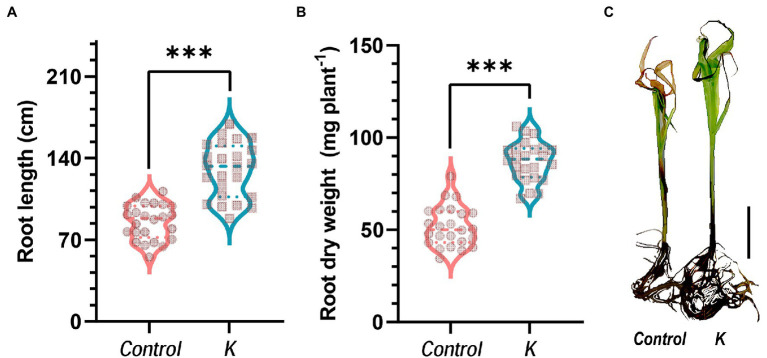
*Mucilaginibacter* sp. K improves maize growth in salt stress. Violin plot showing strain K effects on root length **(A)** and root dry weight **(B)** of 28-day-old maize seedlings under salt stress (100 mM NaCl). Asterisks indicate the statistically significant differences compared to controls (***p < 0.001). **(C)** Morphological comparison of maize seedlings 28 days after growing in a glass tube experiment under gnotobiotic conditions with salt stress (initiated at day 14) in a growth chamber. Scale bar represents 5 cm.

In the glass tube experiment, our earlier study (Fan and Smith, 2021) showed that *Mucilaginibacter* sp. K induced significant increments of root dry weight (unstressed) and shoot dry weight (salt-stressed). In this study also, we observed 1.5- and 1.7-fold increases in the length and dry biomass of seedling root, respectively, in response to *Mucilaginibacter* sp. K as compared with the control, under salt stress (Figures 3A,B).

### Endophytic Colonization

Petri plate counts from roots of inoculated seedlings resulted in bacterial colonies on selective KB plates, but none were observed from plates of control plants. *Mucilaginibacter* sp. K was recovered from inside the roots, indicating that it had colonized the maize roots endophytically, at *ca.* 4.5 × 10^6^ CFU g^−1^ FW. The 7-day-old seedlings were used for this experiment; hence, population size at plant maturity may well have been larger.

### *Mucilaginibacter* sp. K Ameliorates Salt Toxicity

**Figure 4:**
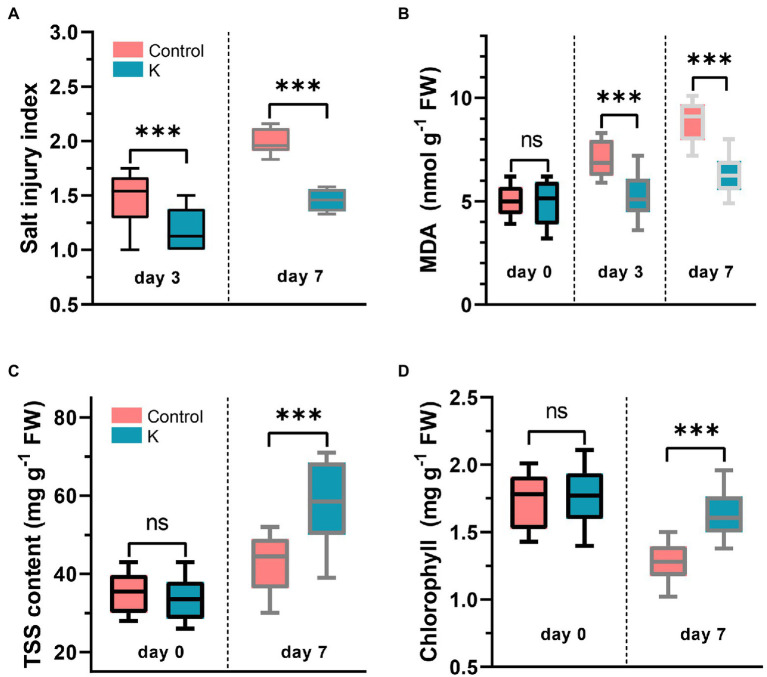
Box plot showing Mucilaginibacter sp. K effects on salt injury index **(A)**, malondialdehyde (MDA) **(B)**, total soluble sugars (TSS) **(C)**, and chlorophyll content **(D)** in leaves of 14-day-old maize seedling (day 0) under salt stress (100 mM) for 3-7 days. The group median and interquartile range were displayed. Asterisks indicate the statistically significant differences compared to controls (***p < 0.001).

Maize leaves were examined for changes of visual (color and turgor) quality over a 7-day period after NaCl treatment. Salt injury index showed a considerable increase from NaCl compared with the control (Figure 4A), in which the seedlings started to slightly curl and brown on leaf edges after day 3, whose symptoms were accelerated over further observation (Figure 3C).

Lipid peroxidation gradually increased during the first 7 days after NaCl treatment (Figure 4B). The initial levels of MDA were not significantly different between the control and stain K, while the concentration of MDA was significantly reduced on day 3 and was still downregulated on day 7 in treated plants. MDA content in strain K-treated leaves was 1.5-fold lower (6 nmol g^−1^ FW) than in control group (over 9 nmol g^−1^ FW) at day 7.

Under nonstress conditions, there were no significant differences of TSS and chlorophyll content between the control and strain K treatment. The TSS content in strain-K treated plants was significantly higher, with a 1.3-fold increase, when compared with the untreated control, under salinity stress (Figure 4C). The total chlorophyll content in salt stressed leaves was dramatically decreased in the control, but strain K inoculation counteracted the loss of chlorophyll and maintained its content in salt-treated plants comparable to control levels (Figure 4D). However, there were no significant differences between the control and treated plants regarding total protein, proline, and total antioxidant capacity (the DPPH assay; Supplementary Figure S1).

### Enzyme Activities

**Figure 5:**
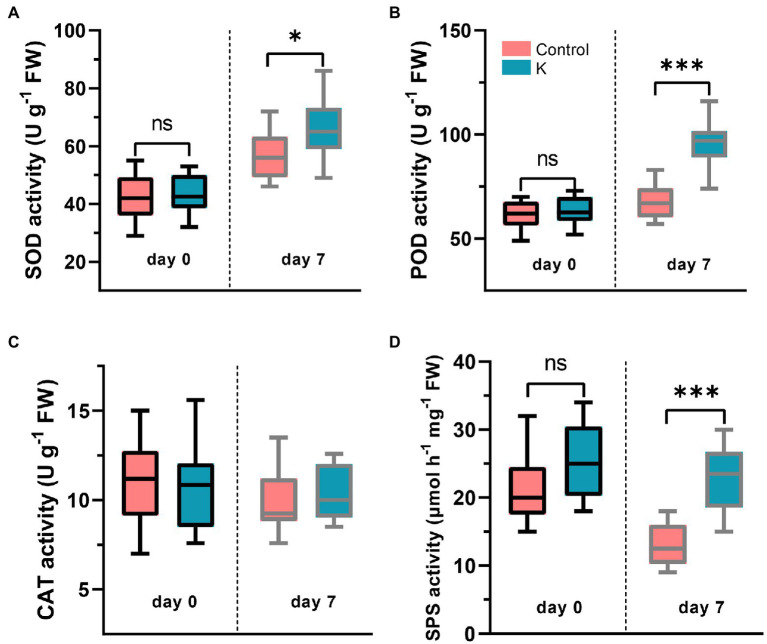
Box plot showing changes of SOD **(A)**, POD **(B)**, CAT **(C)**, and SPS **(D)** activities in maize seedlings treated with *Mucilaginibacter* sp. K or not. The 14-day-old maize seedlings (day 0) were grown under salt stress (100 mM) for up to 7 days, and the 21-day-old seedlings were harvested for enzyme activity analysis (*p < 0.05 and ***p < 0.001).

The activities of ROS-detoxifying enzymes were measured, showing differential responses in maize treated with *Mucilaginibacter* sp. K. Under normal conditions, the activities of CAT, POD, and SOD were not different between strain K inoculation and the control treatments (Figures 5A–C). While bio-primed plants exposed to NaCl showed noticeably higher activities of SOD (by 15.3%; Figure 5A) and POD (by 41.6%; Figure 5B) than the non-primed salt-treated only plants.

The activity of SPS involved in sucrose metabolism was also examined. Under non-stress conditions, no significant variation was observed between strain K-treated plants and the control (Figure 5D). However, there was a decrease in SPS activity in control plants at 100 mM NaCl, while in the presence of bacterial inoculation, plants exhibited an increase (*ca.* 70%) in SPS activity, compared to the controls (Figure 5D).

### *Mucilaginibacter* sp. K Reduces ROS Induced by NaCl

**Figure 6:**
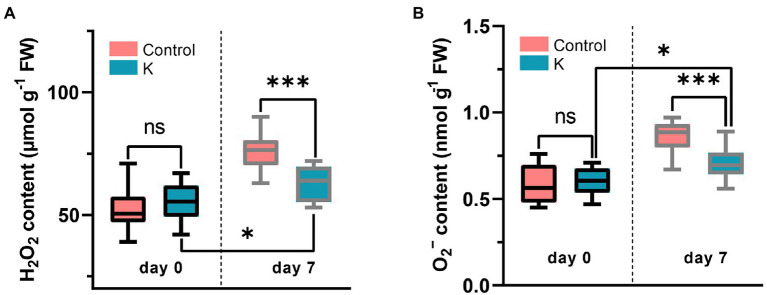
Box plot showing the quantification of H_2_O_2_ **(A)** and •O_2_^-^ **(B)** in maize leaves grown for 21 days in glass tubes under saline conditions (7 days duration). Asterisks indicate the statistically significant differences compared to controls (*p < 0.05 and ***p < 0.001).

The impact of strain K on oxidative toxicity induced by salt stress was assessed by the quantification of the H_2_O_2_ and •O_2_^−^ contents in maize leaf tissue. Maize inoculated with strain K had significantly lower contents of these two ROS, say, H_2_O_2_ reduced by 17.1% and •O_2_^−^ by 18.6%, respectively, as compared with the control (Figure 6), indicating that strain K helped maize seedlings to minimize the NaCl-induced accumulation of ROS.

### Effect of *Mucilaginibacter* sp. K on Ionic Homeostasis

**Figure 7:**
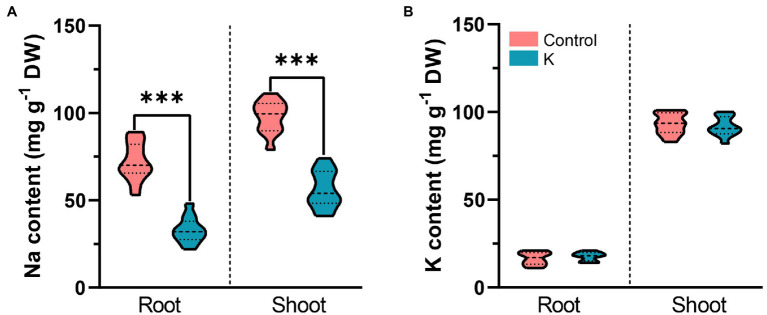
Violin plot for levels of Na^+^ and K^+^ in shoots and roots of 21-day-old maize seedlings inoculated with *Mucilaginibacter* sp. K or not. Details as described in Figure 5. **(A)** Strain K triggers reduction of Na^+^ accumulation in shoots and roots compared with the control. **(B)** Strain K does not affect K^+^ accumulation in maize seedlings. Asterisks indicate the statistically significant differences versus controls (***p < 0.001).

Na^+^ and K^+^ were measured after 100 mM NaCl exposure to decipher the status of ionic distribution between control and strain K-pretreated maize seedlings. Na^+^ content in roots of strain K-treated plants was reduced by 60% compared to control plants on day 7 (Figure 7A). A similar trend was noted in plant shoots, wherein strain K remarkably reduced the total Na^+^ content in leaves by 50% compared with the control (Figure 7A). However, no significant variation was detected in either root or shoot K^+^ level between strain K-treated plants and control under salinity stress (Figure 7B).

### Endogenous ABA Content

**Figure 8:**
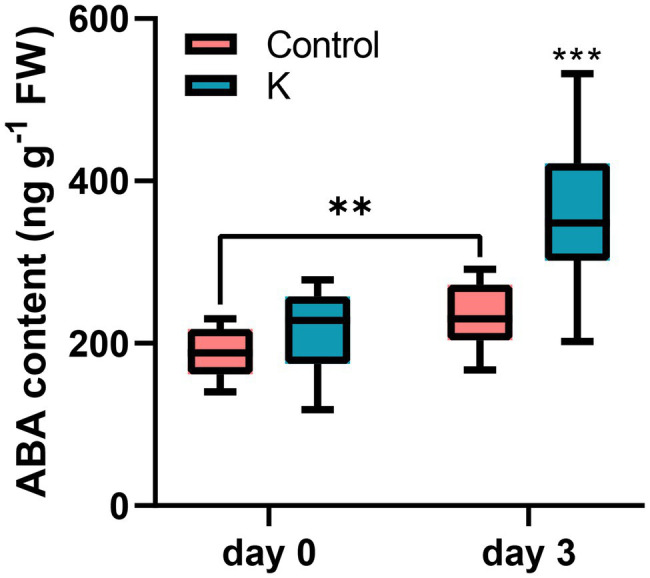
Effect of *Mucilaginibacter* sp. K on endogenous ABA content. The measurements were taken on 14-day-old seedlings (day 0) and 3 days after salt initiation. The increasing ABA content in salt-stressed maize seedlings pre-treated with strain K is greater than that in the control. Box plots show group median and interquartile range (**p < 0.01 and ***p < 0.001).

We previously found that RD29A transcriptional level was significantly induced under salt stress by strain K in Arabidopsis, indicating that ABA might play an important role for salt stress signaling by strain K. Thus, here we measured the endogenous content of ABA in maize. When maize seedlings were grown under normal condition, no difference in the ABA level of seedlings was observed between strain K pretreatment and the control (Figure 8). In salt stressed seedlings, the ABA content was increased; inoculation with strain K further potentiated this increase by 78% compared to the control, after 3 days (Figure 8).

### Effect of *Mucilaginibacter* sp. K on Transcript Levels of Maize

**Figure 9:**
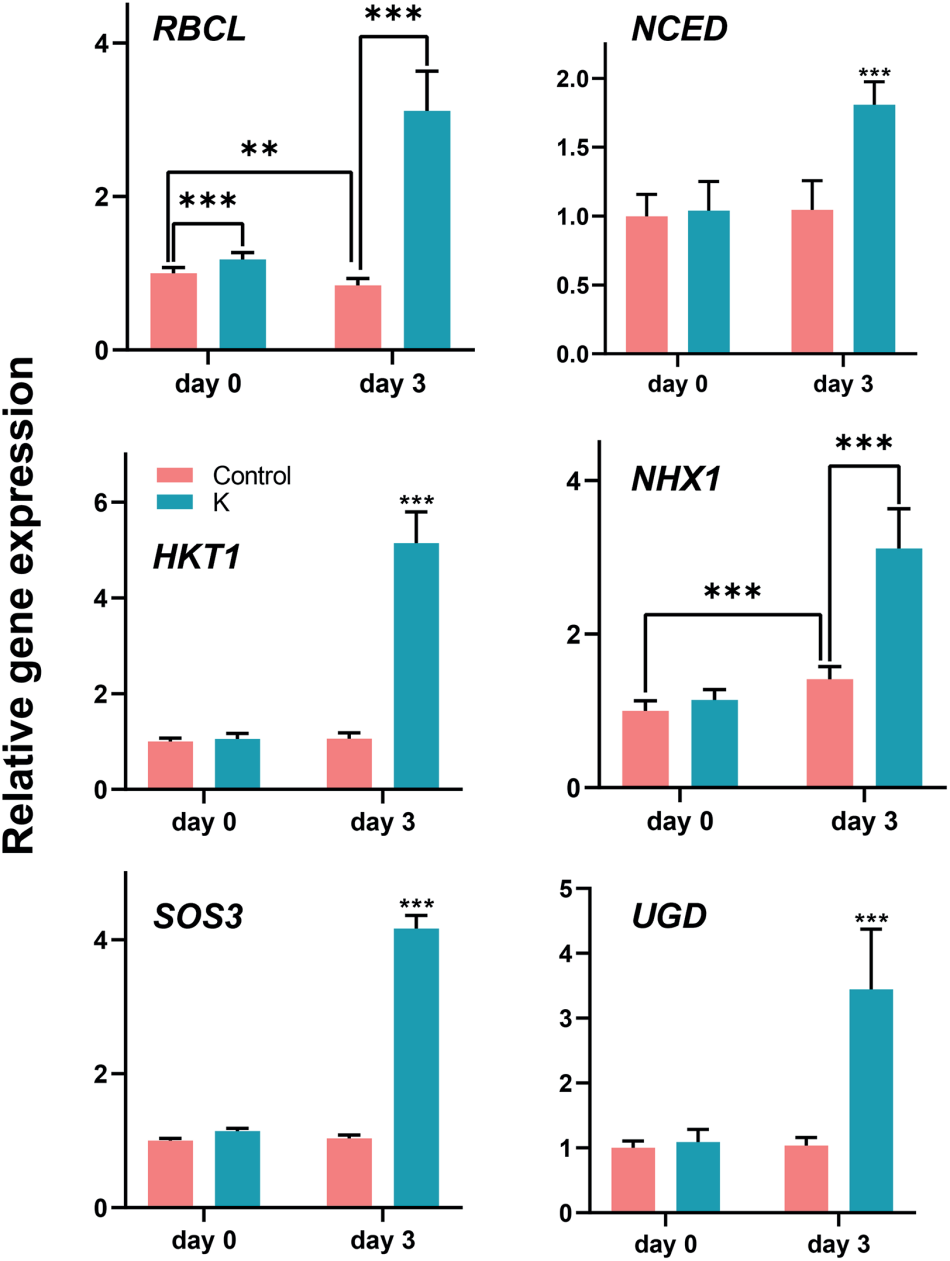
Box plot showing changes of gene expression levels (fold differences) in maize under salt stress. The 14-day-old seedlings (seed bio-primed) were treated with 100 mM NaCl. After 72 h, leaves were collected and subjected to RNA isolation, followed by quantitative real-time reverse transcription-PCR. Asterisks represent values statistically different from the control at **p < 0.01 and ***p < 0.001.

To further elucidate the role of strain K in ameliorating plant growth under salt stress, the expression of genes related to ion homeostasis and cell growth was evaluated in maize leaves under normal conditions and after 3 days of salt treatment (Figure 9). The expression of *RBCL* (encoding ribulose-1,5-bisphosphate carboxylase/oxygenase large subunit) was significantly enhanced in seedlings grown with strain K for 14 days. After 3 days of imposition of salt stress, the transcription of *RBCL* was significantly reduced in control seedlings but was upregulated (*ca.* 4-fold) in those pretreated with strain K. Under salinity stress, the expression level of *NCED* (a key enzyme in abscisic acid biosynthesis) was approximately 1.78 times greater with the inoculation of strain K than that in the control, further confirming that *Mucilaginibacter* sp. K could stimulate ABA accumulation and regulate ABA signaling pathway.

Expression profiles of genes involved in ionic transporters revealed similar patterns in strain K-treated seedlings under stressed conditions (Figure 9). Enhanced expression of *HKT1* (encoding high-affinity K^+^ transporter 1; 4.9-fold higher), *NHX1* (encoding Na^+^/H^+^ antiporter; 2.2-fold higher), and *SOS3* (a Ca^2+^ sensor protein; 4-fold higher) was observed in bio-primed seedlings as compared with untreated controls under salt stress, while it remained unchanged under unstressed conditions. In case of *NHX1*, salt stress also resulted in a 1.4-fold increase over the control seedlings. The transcription of *UGD* was increased approximately 3.4-fold in bio-primed and salt-treated seedlings, indicating strain K-mediated plant growth under salinity might involve carbon metabolism (cell wall synthesis).

### Maize Growth Under Pot (Controlled Environment) Conditions

#### Promotion on Plant Growth and Biomass Production

The effect of *Mucilaginibacter* sp. K on the greenhouse growth of maize plants under initially nonsterile conditions was studied. There were no significant differences in the height of maize plants (Table 2A). Inoculated plants had numerically larger leaf area and circumference, though the values of these variables were not statistically different from those of the controls. Treatments with strain K improved root dry weight per maize plant by 22.78%, compared to the control. The same treatment also resulted in greater root length, with an increase of about 37% (Table 2A).

**Table 2:** Effect of *Mucilaginibacter* sp. K on the growth parameters (A) and biomass (B) of maize plants at 35 DAS.

**(A)**				
	**Height (cm)**	**Circum ference (cm)**	**Root length (cm)**	**Leaf area (cm** ^**2**^ **)**
Control	73	3.02	571	663.46
K	76.6	3.30	778.10***	814.57
**(B)**				
	**Aerial biomass (g)**	**Aerial dry weight (g)**	**Aerial dry matter (%)**	**Root dry weight (g)**
Control	33.06	3.8	11.74	1.228
K	37.86	4.44	11.66	2.129***

*** *Indicates differences significant at p < 0.001.*

A significant difference was also observed between the effects induced by rhizobacterial strain K for biomass production by maize plants (Table 2B). At 35 DAS, strain K increased root dry weight by 73.37%. Though the fresh aerial biomass and above-ground dry weight was not significantly altered, there was an increasing trend in strain K-treated plants.

#### Total Carbohydrate, Proline Content, and Antioxidant Activities in Maize Leaves

Total soluble carbohydrate content was 4.2 mg g^−1^ DW in the control plant, strain K treatment increased carbohydrate content in maize leaves by 37.6% compared with the control (Supplementary Figure S2A). Generally, inoculation with strain K did not significantly influence the antioxidant activities in maize leaves, regarding total phenolics and scavenging effect against DPPH radicals (Supplementary Figures S2B,C). Our findings also indicated that plants synthesized similar proline level between strain K-treated and non-inoculated controls (Supplementary Figure S2D).

#### Acquisition of Nutrient Content by Maize

Inoculation of strain K not only enhanced maize growth but also influenced nutrient uptake by maize plants at 35 DAS (Table 3A). Strain K notably increased the N levels in the aerial biomass of maize plants, by 28.7%, over the non-inoculated control. However, the other macro- and micronutrients were not significantly influenced by strain K, as compared with untreated controls; the same also hold true for N/C ratio.

**Table 3:** Influence of rhizobacteria on nutrient content and C and N composition (dry weight basis) of maize leaves (A) and roots (B) at 35 DAS.

Treatment	P	K	Mg	Ca	Na	Fe	Cu	Carbon (%)	Nitrogen (%)	N/C
	(mg g^−1^)	(mg g^−1^)	(mg g^−1^)	(mg g^−1^)	(μg g^−1^)	(μg g^−1^)	(μg g^−1^)			
**(A)**										
Control	1.01	27.1	1.99	2.7	8.13	40.59	2.76	50.4	1.29	0.026
K	1.20	26.1	1.76	2.56	6.25	32.46	2.12	50.6	1.66^*^	0.033
**(B)**										
Control	0.43	8.96	0.71	1.2	245.14	149.09	5.48	47.2	1.19c	0.025
K	0.45	9.22	0.88	1.48	374.17	182.9	4.82	46	2.52^***^	0.055

* *Indicates differences significant at p < 0.05.*

*** *Indicates differences significant at p < 0.001.*

There were also some differences in the concentrations of nutrients in the roots, when compared to those in shoots (Table 3B). In line with those in leaves, all the tested minerals in the roots were not significantly influenced by bacterial treatment, except that the N content was increased up to 112%, with treatment by strain K.

## Discussion

*Mucilaginibacter* sp. K is a plant growth-promoting strain and salinity stress alleviator as ascertained in *A. thaliana* (Fan et al., 2020). Seedling establishment, a key determinant for yield, is severely impacted in maize by salinity stress. In the current study, we investigated growth enhancement (greenhouse trials) as well as induced priming for effects on salinity resistance by inoculation with strain K at the signaling (in Arabidopsis), and physiological, molecular, and metabolic levels (in maize seedlings).

Mitogen-activated protein kinase (MAPK) cascades in Arabidopsis can be activated by diverse intra- and extracellular stimuli including hormones and cellular stressors, and they act as signaling regulators involved in immune responses as well as fundamental physiological processes (Komis et al., 2018). The stimuli-induced activation of MAPK is liable for the activation of transcription factors, resulting in the induction of defense-related genes (Khan et al., 2020). MPK6 played an important role in root hair development induced by *Trichoderma atroviride* (Contreras-Cornejo et al., 2015). MPK6 was also shown to be engaged in leaf colonization and growth promotion by *T. viride* Tv-1511 in Arabidopsis (Guo et al., 2020). Previously, we demonstrated that *Mucilaginibacter* sp. K promoted growth in Arabidopsis under both normal and saline conditions (Fan et al., 2020), and suggested that growth promotion effect of strain K in Arabidopsis was dependent on MPK6 since *mpk6* mutant but not *mpk3* seedlings impaired strain K-induced increases of fresh weight and leaf area at day 21 after seed inoculation (Fan and Smith, 2021). At the transcription (Fan and Smith, 2021) and protein activity levels (Figure 1C), we found that MPK6 activity could be increased by strain K under unstressed conditions, confirming that strain K-mediated growth promotion is dependent on MPK6. MPK6 could be activated by salt (Zhou et al., 2022) and mediated salinity stress response following sequential phosphorelay (Yu et al., 2010). Although the accumulation of mRNA of *MPK3* and *MPK6* was not significantly influenced by strain K 12 h after salt treatment (Fan and Smith, 2021), the present work revealed a critical role of MPK6 in strain K-induced salt response signals, wherein *mpk6* mutant but not *mpk3* impaired salt tolerance by strain K (Figure 1D). These results suggest that strain K induces growth/stress-related signals to MPK6 or to other unknown components to activate MPK6, bringing about enhanced growth promotion and salt tolerance in plants.

Phytohormones act critically in plant defense, development, and growth regulation. Through the complex cross talk within the signaling cascades evoked by these growth regulators, they can fine-tune the expression of genes important to plant growth and development (Khan et al., 2020). To analyze hormone signaling essential for growth promotion under normal and saline conditions in response to *Mucilaginibacter* sp. K, we used an Arabidopsis transgenic line and four mutant lines, along with three phytohormone biosynthesis inhibitors. Among the four selected phytohormones, ethylene (ET) and salicylic acid (SA) are regarded as stress response hormones while auxin and gibberellin (GA) as growth promotion related (Verma et al., 2016). NPR proteins are known to be involved in SA-mediated stress responses. Our growth promotion evidence revealed the involvement of some phytohormones in a strain K-mediated signaling cascade. The mutant line *eir1* showed no growth regulation effect exerted by strain K (Figure 2A). Under salt stress, growth enhancement was abolished in neither *eir1* nor *gai1* (Figure 2B). AVG and DZ treatment diminished the growth stimulation effect by strain K (Figure 2C). The growth enhancement by strain K in *npr1*, *etr1*, and NahG plants suggests that strain K ET- and SA-independently benefits plants under normal and saline conditions. Our data also revealed that the auxin signaling pathway is vital to growth promotion in inoculated plants under non-stressed conditions, while the auxin and gibberellin pathways are necessary for strain K-mediated salt tolerance. This is in line with the findings where the priming effect of *A. faecalis* JBCS1294 on salt stress in Arabidopsis is dependent on the auxin and GA pathways (Bhattacharyya et al., 2014). Plant’s endogenous auxin signaling can be triggered by PGPR, leading to cell growth stimulation (Spaepen and Vanderleyden, 2011). Correspondingly, our earlier study showed that expression of auxin-induced *At4g36110* gene was upregulated by strain K after salt exposure, indicating that this strain might alleviate salt stress *via* altering endogenous auxin content. Exogenous application of gibberellin on citrus leaves reduced ROS-mediated cell death and Huanglongbing symptoms (Ma et al., 2022). We previously found that the expression of the *GA3ox1* gene involved in GA biosynthesis was induced by strain K (Fan and Smith, 2021), indicating that GA might directly ameliorate oxidative damage to mitigate salt toxicity. Nevertheless, the study on how these pathways (auxin and gibberellin) cross talk with each other or other signaling, say, MPK6, in strain K-inoculated plants, will throw light on the mechanisms underlying salt tolerance induced by strain K. Moreover, how and by which signaling components these phytohormones and MPK6 signals are generated remain to be explored.

The effect of strain K on crop plants was also systematically investigated. Since Arabidopsis is a dicot, here we chose a monocot, maize, for the present study. In the first part of our investigation, we determined seed germination effects of strain K on maize under *in vitro* conditions. Strain K induced better germination of maize compared with the control, in that shoot and root length, seedling fresh weight, and vigor index were all significantly increased (Table 1). Similar improvement of seed germination variables by PGPR has been reported in rice (Swain et al., 2021) and maize (Nezarat and Gholami, 2009). These findings may be due to better synthesis of endogenous plant growth regulators, i.e., auxins, gibberellins, and cytokines, which can stimulate cell division and elongation or trigger specific enzymes assisting in starch assimilation (Subramanian et al., 2016).

Strain K inoculation improved seedling growth (Fan and Smith, 2021) under sterilized and non-sterilized (present study) conditions. Since strain K lacks many of the PGP traits, such as N_2_-fixation, phosphate solubilization, and HCN and siderophore production (Fan and Smith, 2021), growth promotion in maize by strain K might be mediated by other mechanism(s), other than the commonly recognized PGP traits. Total N content was statistically greater in strain K-treated maize under greenhouse conditions than the control (Table 3), indicating that agronomically significant levels of N were probably not primarily supplied through the N_2_-fixation, suggesting that (1) there may be alternate mechanisms implied by strain K that are more important than contributions through N_2_-fixation, (2) the increase in root length (Table 2A) may have contributed to the increased N content in maize leaves (Table 3A) as the two variables were significantly correlated (*R*^2^ = 0.71), and (3) strain K introduction might positively affect environmental microbiome, which in turn played important roles in N assimilation. It will be intriguing to employ omics technologies to study the regulatory role of strain K in the soil microbial community as well as the network in plant-strain K interactions. Photosynthesis is one of the rate-limiting points that determine plant growth since it offers fixed carbon for energy, growth, and production. The photosynthetic rate of maize in greenhouse plants inoculated with strain K was slightly but significantly enhanced on 21 and 35 DAS (data not shown), suggesting that strain K might increase maize growth and production through higher photosynthetic activity; while strain K did not increase the photosynthetic rate in cannabis grown in an indoor facility until the mid-point for flower formation (Lyu et al., 2022), indicating that the plant growth stages, conditions, or plant species might influence the effect of strain K on photosynthesis.

IAA is one of the mechanisms that PGPR use to promote plant growth; this mechanism regulates several aspects of plant growth and development such as lateral root initiation, cell enlargement, cell division and increased root surface, leading to increased N-uptake due to hormonal effects on root morphology and activity. It should be noted that although we detected endogenous IAA production in strain K (Fan and Smith, 2021), there is uncertainty regarding the importance of this mechanism. We cannot conclude that bacterial auxin secreted by strain K plays a key role in plant fitness unless the mutant bacterial strain has been tested (Tzipilevich et al., 2021). Strain K also ameliorated salinity stress in regard to root dry weight and root length (Figures 3B,C). Moreover, *Mucilaginibacter* sp. K efficiently colonized the interior parts of Arabidopsis (whole seedlings; Fan et al., 2020) and maize roots (present study). This genus has been shown to persist and dominate in roots of gray poplar (Fracchia et al., 2021). These findings indicated the possibility of yield increase and low inputs in maize by strain K, since strengthened root system is indispensable for potential nutrient and water uptake (Qin et al., 2006).

PGPR can use a range of strategies to reduce the vulnerability of plants to environmental stresses. Salinity exacerbated membrane lipid peroxidation which has often been used as one of the salinity stress biomarkers for increased oxidative damage. In our findings, the attenuated increase in MDA accumulation in strain K-treated maize seedlings implied that the oxidative damage might be counteracted by the upregulated activities of antioxidant enzymes, i.e., POD and SOD, in the leaves (Figure 5), suggesting that strain K stimulates plant antioxidant defense systems of maize for ROS scavenging under salt stress. In line with this observation, lower H_2_O_2_ and •O_2_^−^ contents were found in strain K–treated seedlings compared to control (Figure 6), which might be explained by the increased antioxidant enzyme activities that protected maize from ROS burst and the subsequent photosynthetic impairment, leading to normal cell function.

Chlorophyll is critical in photosynthesis for crop production. Kandasamy et al. reported that *Pseudomonas fluorescens* enhanced expression of ribulosebisphosphate carboxylase large chain precursor, which plays a principal role in chlorophyll accumulation and photosynthesis (Kandasamy et al., 2009). The higher *RBCL* expression (Figure 9) and lower decrease in total chlorophyll content in seedlings treated with strain K suggest that this bacterium may help maintain photosynthetic capacity in maize under saline conditions, a potential mechanism used by strain K to cause growth promotion.

Plant also suffered from osmotic stress under saline conditions. Reports have shown that PGPR could stimulate the accumulation of osmoprotectants, i.e., polyols, amino acids, and betaines, in plants to counteract osmotic stress by stabilizing subcellular structures, scavenging free radicals and protecting against dehydration (Munns, 2002). A *Pseudomonas* sp. S3 isolated from turmeric rhizosphere enhanced proline content and profoundly increased TSS in tomato under salt stress (Pandey and Gupta, 2020). Increased levels of TSS in maize inoculated with *Pseudomonas* spp. correlated with better drought stress tolerance (Sandhya et al., 2010). Our data showed that the content of TSS but not proline was significantly induced by strain K to combat salt stress (Figure 4), indicating that the osmotic adjustment is at least partially account for salt tolerance exerted by strain K treatment in maize seedlings. Though the accumulation of TSS and proline was not affected under normal conditions (Figure 4C) in our study, there has been some controversy about proline as an indicator of stress responses by other researchers. For instance, proline content in wheat was increased significantly under salinity stress by inoculation with *Dietzia natronolimnaea* (Bharti et al., 2016) but reduced by *Pantoea alhagi* (Chen et al., 2017). Inoculation with PGPR significantly enhanced the proline content of wheat under both non-saline and salt stress conditions (Singh and Jha, 2016). By contrast, Rojas-Tapias et al. showed an increase in proline contents due to treatment with PGPR under normal conditions, along with a decrease in proline concentration in maize leaves under salinity stress (Rojas-Tapias et al., 2012). These data indicated that PGPR might invoke some biotic stress responses, which triggered proline biosynthesis in plants or perhaps suppressed the NaCl-induced damage through an alternative strategy for the activation of enzymatic and non-enzymatic organic antioxidants/osmolytes such as glycine betaine, SOD, CAT, etc. (Kim et al., 2017), and this might apply to our case, where strain K induced SOD and POD activities. Studies on the accumulation of glycine betaine and reduced glutathione (non-enzymatic antioxidant) mediated by strain K could confirm this assumption.

Besides the potential protecting effects on photosynthesis, osmotic stress, and ROS toxicity, a question was raised regarding whether this *Mucilaginibacter* sp. helps maize seedlings overcome Na^+^ toxicity *via* ion redistribution. Excessive Na^+^ in plants could disrupt K^+^/Na^+^ balance and ultimately disturb plant metabolism and productivity through damaging the photosynthetic apparatus (Zhong et al., 2020). Our study showed that salinity dampened ion homeostasis in that Na^+^ accumulation in both root and shoot increased under stressed conditions in controls; whereas in strain K-treated seedlings, the increase in Na^+^ content upon salt stress was disrupted (Figure 7A), asking whether strain K reduces internal Na^+^ through exclusion, excretion and/or compartmentalization. PGPR have been shown to help in mediating ion transportation. In higher plants, HKT1 is a Na^+^ transporter that mediates Na^+^ homeostasis. *AtHKT1* expression in shoot was significantly triggered by *Bacillus subtilis* GB03 (Zhang et al., 2008). *ZmHKT1* null was highly sensitive to salt and increased leaf Na^+^ accumulation (Zhang et al., 2018). Our results indicate that the expression of *HKT1* gene was significantly induced 3 days postexposure to salt in the shoot of strain K-pretreated seedlings (Figure 9), indicating strain K could facilitate in selective recirculation of Na^+^ from shoots; this is of great importance since leaf Na^+^ balance is entwined with salt tolerance in maize. The expression of another gene, *NHX1*, located in the tonoplast and controls Na^+^ export and sequestration, was also upregulated in control under salt stress, while strain K-treated seedlings increased to a greater extent (Figure 9). This result implied that strain K could promote Na^+^ compartmentalization, thus mitigating cytosolic Na^+^ toxicity. NHX1 might also contribute to osmotic stress alleviation by strain K during salinity exposure in that it has been shown to function in recruiting solutes for water uptake under salt stress (Adabnejad et al., 2015). We also found that the transcription level of *SOS3* was noticeably induced in strain K-pretreated seedlings under salt stress (Figure 9). The function of SOS3 is dissimilar to that of Na^+^/ H^+^ antiporters in that, after Ca^2+^ signal perception, SOS3 activates SOS2, which in turn forms a kinase complex with SOS3 and subsequently regulate ion transporters such as NHX1 and SOS1 (Chinnusamy et al., 2006), leading to possible maximum of cellular Na^+^ efflux. It has been shown that overexpression of *ZmSOS3* in Arabidopsis leads to increased salt tolerance, reduced Na^+^ content, and induced *AtNHX8* expression (Wang et al., 2007b). Taken together, these results demonstrated that the promoted expression of genes, namely, *HKT1*, *NHX1*, and *SOS3*, involved in Na^+^ excluding, recirculating and sequestering, and Ca^2+^ perception, might contribute to salt tolerance in maize seedlings by strain K; a similar pattern was also observed in Arabidopsis treated with *Burkholderia phytofirmans* PsJN (Pinedo et al., 2015). Mutant plants with impaired HKT1, NHX1, or SOS3 could be applied to confirm the involvement of these genes in strain K-induced salt tolerance. Moreover, salt-tolerant maize inbred line exerted faster induction of genes related to Ca^2+^ transport (Gao et al., 2016), thus whether strain K could also upregulate these genes is worth studying since Ca^2+^ is an essential messenger for early cellular responses to various stresses, including salinity (Xu et al., 2022).

In greenhouse experimentation, strain K decreased Na shoot concentration, along with significantly lowering the Na^+^/K^+^ ratio when compared to non-inoculated plants under non-stressed conditions (Table 3A), indicating that this strain might assist in reducing salt toxicity through lower Na/K ratios in shoots; this is considered as a predominant mechanism for enhancement of plant growth under salt stress (Munns and Tester, 2008). And indeed the *in vitro* experiment showed that strain K reduced Na^+^ content in maize seedlings and ameliorated salt stress (Figure 7). Similar observations were reported for crops such as inoculated peanut (Sharma et al., 2016) and maize (Abd El-Ghany et al., 2015). It is worth noting that exopolysaccharide (EPS) produced by strain K (Fan and Smith, 2021) might play a role in alleviating salt stress since EPS has been shown to bind Na^+^, thus decreasing Na^+^ availability for plant uptake (Ashraf et al., 2004). It should be noted that the Cl^−^ toxicity was not evaluated in the present study and it would be worthwhile to conduct additional experimentation in this area.

As an important phytohormone, ABA has been shown to contribute to salt tolerance in PGPR-treated plants, and its biosynthesis is closely associated with stress resistance in plants (Muppala et al., 2021). *Dietzia natronolimnaes* STR1 potentiated salinity tolerance in wheat through upregulation of an ABA signaling cascade and modulation of expression of genes related to ion transport and antioxidant enzymes (Bharti et al., 2016). Exogenous application of a functional analog of ABA alleviated salt stress in maize *via* enhanced root growth, increased antioxidant enzyme activity, and upregulated expression of aquaporin genes (Geng et al., 2021). We previously found that strain K inoculation led to higher transcription of the ABA-dependent *DR29A* following salinity stress in Arabidopsis (Fan and Smith, 2021), showing that strain K might prime for ABA-dependent signaling upon salt stress. In the current study, the expression of *NCED*, a key gene encoding rate-limiting ABA biosynthesis enzyme, in maize seedlings treated with strain K, was induced (Figure 9). Moreover, ABA content in maize leaves was significantly increased upon salt treatment as compared with the control (Figure 8). A similar phenomenon was caused by volatile compounds from *Rahnella aquatilis* JZ-GX1 in Arabidopsis under iron deficiency (Kong et al., 2021). Co-transformation of *NCED* with *RPK* mitigated drought stress in maize, in terms of growth as well as physiological and biochemical variables tested, such as increased total chlorophyll and ABA content under stress condition (Muppala et al., 2021). From previous research it has been shown that ABA could upregulate transcriptional levels of MAPKs; ABA also could increase cytosolic Ca^2+^, engendering the upregulation of SOS3 and the subsequent activation of SOS3-SOS2 regulatory pathway (Jannesar et al., 2014). Taken together, our result suggest that strain K might induce maize resistance to salt stress through ABA signaling. The faster ABA production (Figure 8) and expression of ABA-regulated genes (Fan and Smith, 2021) in strain K-induced salt stress could probably lead to attenuated dehydration, thus proline accumulation is not needed. The increase in ABA content in control maize plants under salt stress was inhibited by *Bacillus amyloliquefaciens* SQR9 (Chen et al., 2016). The contradiction here might be due to (1) different signaling pathways employed by different PGPRs, and (2) antagonistic interactions between ABA-dependent and ABA-independent signaling employed by plants treated by growth regulators. As a central regulator to various abiotic stress responses, ABA signaling might form sophisticated cross-talks with other phytohormones and signaling pathways, say, GA, auxin, SOS3, and MPK6 in the present work, to modulate strain K-mediated salt stress responses in plants, leading to an optimal growth under this specific environmental condition. However, further experiment using mutant plants that are ABA deficient or insensitive would be ideal to determine whether ABA-dependent signaling is responsible for strain K-induced salt tolerance.

AtSPS1F has been reported as one of the downstream targets of MPK6 in Arabidopsis, especially upon stress perception (Rayapuram et al., 2017); MPK6 can facilitate the dephosphorylation of SPS1F, leading to its activation. AtSPS1F is expressed in mature leaves and is one of the major functional isoforms in leaf carbohydrate metabolism (Volkert et al., 2014). Overexpression of maize SPS in potato significantly delayed leaf senescence and increased leaf SPS activity (Ishimaru et al., 2015). The incremented SPS activity (Figure 5) observed in the current study might partially contribute to the expanded period of photosynthesis, leading to better performance of plants under saline conditions. An increase in SPS activity increased the photo assimilatory supply and improved tuber yield in the transgenic potato (Ishimaru et al., 2015), leading to the possibility that strain K might increase yield of maize as well. Further study on maize yield, following strain K inoculation under both unstressed and saline conditions is needed to test this assumption.

There have been several reports showing that bacterial inoculation induced synthesis of carbohydrate in maize (Subramaniyan et al., 2012; Abd El-Ghany et al., 2015) in agreement with the current study where inoculation with strain K resulted in higher total soluble carbohydrate (*ca.* 7.0 mg g^−1^) than control plants (*ca.* 4.0 mg g^−1^; Supplementary Figure S2). UDP-glucose dehydrogenases (UGD) catalyze UDP-glucose conversion to a precursor of pectin, UDP-glucuronic acid. UGD plays an important role in the synthesis of pentose of cell walls in maize, and subsequently the cell growth (Karkonen et al., 2005). Overexpression of an UGD from cotton significantly elongated the root of transgenic Arabidopsis (Jia et al., 2021). Here, we found that the gene expression of *UGD* in maize was significantly accreted by strain K (Figure 9), indicating the possibility that strain K stimulates maize growth through root elongation and cell wall synthesis. Studying the growth promotion effect of strain K using *UGD* mutant plants under both normal and salt stress conditions will confirm whether this gene is involved in the growth stimulation effect of strain K.

## Conclusion

**Figure 10:**
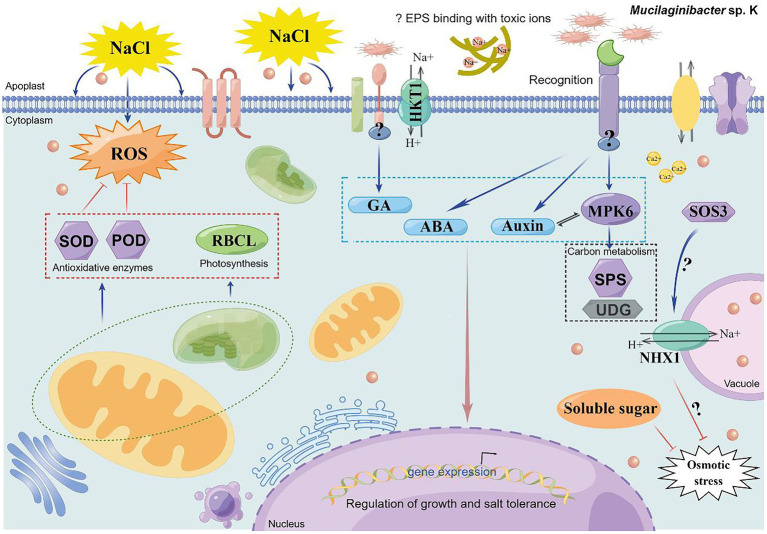
A schematic proposed model of *Mucilaginibacter* sp. K-mediated salt tolerance in plants [drawn by Figdraw (www.figdraw.com)]. Based on the results from current study, we propose that stain K participates in salt tolerance through activating the GA- and auxin-dependent signaling pathways, which might lead to concurrent expression of genes involved in growth promotion and salinity resistance. Strain K colonizes roots and might enhance photosynthesis through upregulation of chlorophyll content and transcription of *RBCL*. Strain K induces Na^+^ efflux and sequestration to lower ion cytotoxicity. Strain K also enhances activities of antioxidant enzymes such as POD and SOD and soluble sugar content in strain K-treated plants, contributing to reduced ROS degradation and osmotic stress, respectively. Strain K promotes ABA accumulation, which might play a role in the activation of SOS3 through Ca^2+^ flux. Specifically, the growth promotion and salt tolerance conferred by strain K is dependent on MPK6 signaling, which function in regulating ROS homeostasis, ionic homeostasis, and plant growth and development. As downstream targets of MPK6, SPS, and UDG are also positively influenced by strain K in salt-stressed plant, probably leading to enhanced cell growth. ABA signaling might form complex cross-talks with GA, auxin, and MPK6 to modulate strain K-mediated salt stress responses in plants, leading to an optimal growth under this specific environmental condition. This model may explain how the strain K trigger salt tolerance when it is root-tip or seed inoculated.

The current study reports that *Mucilaginibacter* sp. K promoted plant growth, evoked salinity tolerance, and increased cytosolic signal in plants through the auxin, GA, and MPK6 signaling pathways. Strain K downregulated ROS toxicity and upregulated the expression of key genes in ion transportation and carbon metabolism. Seed inoculation of strain K is a sustainable strategy to improve maize growth under saline conditions. Thus, a model of the preliminary mechanisms of strain K-mediated salt tolerance was proposed (Figure 10). The results presented here provide a better understanding of how plants respond to strain K and offer putative targets for further digging into the mechanisms. Though *Mucilaginibacter* has been shown to be one of the more abundant genera in agricultural soils (Dorr de Quadros et al., 2012), to the best of our knowledge, there has been no systematic study regarding PGP effects and mechanisms associated with *Mucilaginibacter* spp. on food crops, except for the current study. As such, this is the first detailed report about the potential agricultural applications of a *Mucilaginibacter* sp. from a wild plant, fall dandelion. Although bacterial growth promotion is sometimes species or genotypic specific, *Muciligbacter* sp. K displayed PGP effects in several crop plants, including canola (Fan, 2018), maize, and cannabis, under non-sterilized indoor environments, suggesting that this strain is very promising as an eco-friendly nutrient and abiotic stress management option for at least greenhouse production. Strain K is also of great potential for enhancing the efficiency of biomass production for sustainable and profitable production of biofuels from maize, particularly if the negative impacts of environmental stressors such as salinity, drought, and infertility can be mitigated upon inoculation. It was unknown whether the benefits of strain K could extend from germination, seedling, and grown-up stages to production stage in maize. As such, a more comprehensive and detailed study should be undertaken to ascertain the extent to which strain K beneficially influences plant growth, crop production, and biofuel production under saline stress-prone field conditions.

## Data Availability Statement

The original contributions presented in the study are included in the article/Supplementary Material, further inquiries can be directed to the corresponding author.

## Author Contributions

DF performed the experiments, analyzed the data, and wrote the original draft of the manuscript. DS supervised the project, provided intellectual input, and edited the manuscript. All authors contributed to the article and approved the submitted version.

## Funding

Financial support was provided by the Canadian Natural Sciences and Engineering Research Council of Canada, number RGPIN-2020-07047.

## Conflict of Interest

The authors declare that the research was conducted in the absence of any commercial or financial relationships that could be construed as a potential conflict of interest.

## Publisher’s Note

All claims expressed in this article are solely those of the authors and do not necessarily represent those of their affiliated organizations, or those of the publisher, the editors and the reviewers. Any product that may be evaluated in this article, or claim that may be made by its manufacturer, is not guaranteed or endorsed by the publisher.

## Footnotes

^1^https://www.ncga.com/ (Accessed: February 22, 2022).
