# Supplementary material for: Mucilaginibacter sp. K Improves Growth and Induces Salt Tolerance in Nonhost Plants via Multilevel Mechanisms
Source: Front Plant Sci. 2022 Jun 27;13:938697. doi: 10.3389/fpls.2022.938697 (PMC9271937; doi:10.3389/fpls.2022.938697)
Supplement: Supplementary file 1 [file Table_1.DOCX]

Supplementary Material


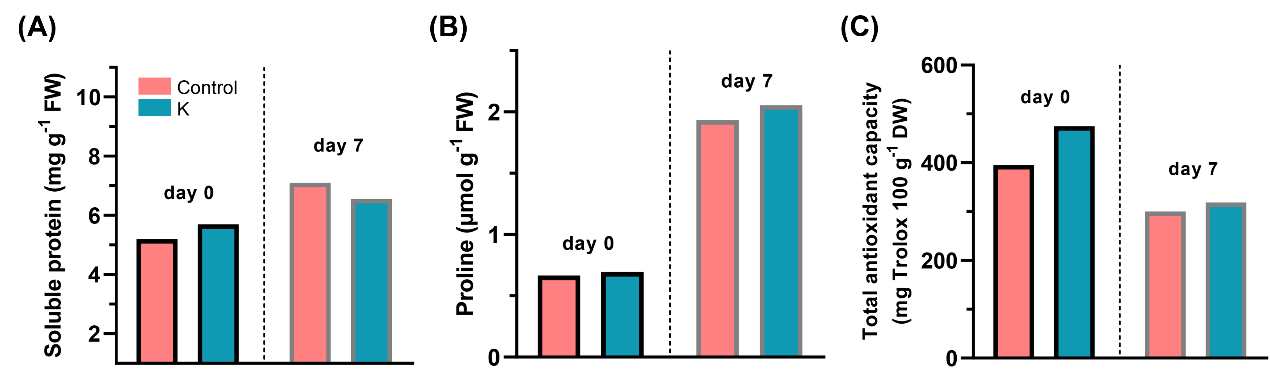


**Fig. S1.** *Mucilaginibacter* sp. K effects on total soluble protein (A), proline content (B), and total antioxidant capacity (reduction of DPPH) in maize seedling shoots after 21 days growth. Salt stress was initiated at day 14. The results are mean ± standard error from three independent experiments.


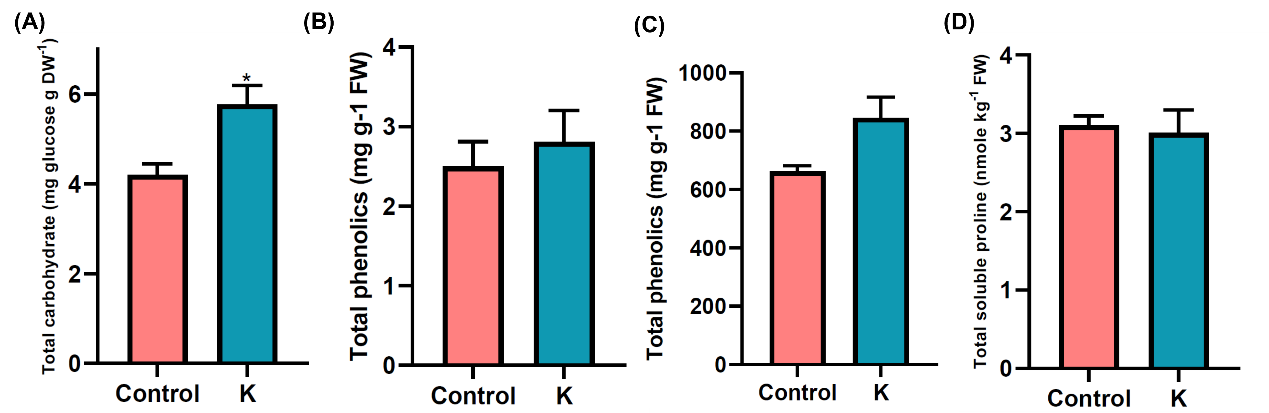


**Fig. S2.** Effect of *Mucilaginibacter* sp. K inoculation on the production of carbohydrate (A), ROS scavenging activity (B), total phenolics (C), and proline content (D) of maize. Sterilized maize seeds were sown into pots and treated with strain K on days 0 and 7. Leaves were harvested at 35 DAS. Asterisk indicates statistical difference by t test analysis with *P* value < 0.05, as compared to control. The experiment was repeated twice.

| **Table S1** | Primer sets used for maize in qRT-PCR study. | | | |
| --- | --- | --- | --- | --- |
| **Gene name** | | **Primer sequences (5’-3’)** | **Reference** |  |
| *HKT1* | | F: TCGGCTCTGGACCTACTCTT | Zhang et al. (2018) |  |
|  |  | R: ACGACGACGACTCTGCTCTA |  |  |
| *RBCL* | | F: GCTGCCGTTGAGGAAGGTATTG | Chen et al. (2016) |  |
|  |  | R: TGCTCGTCGTTCTCCAGTGTAT |  |  |
| *UGD* | | F: GGATCTCCTCTGTGAACGCC | current study |  |
|  |  | R: TTCTGGAAGCAAGACCCACC |  |  |
| *NCED* | | F: CCGCCGACTCCATCTTCAA | Chen et al. (2016) |  |
|  |  | R: TTCACCATCCCGACCTCCA |  |  |
| *NHX1* | | F: ACTTGTTCTTCACCAGCACCATACT | Chen et al. (2016) |  |
|  |  | R: ATTCCACTCAGGTCCAACAGCATT |  |  |
| *UBI2* | | F: TGGTTGTGGCTTCGTTGGTT | Zhang et al. (2018) |  |
|  |  | R: GCTGCAGAAGAGTTTTGGGTACA |  |  |
| *SOS3* | | F: GACTTTCAGGCAAGCGGACA | current study |  |
|  |  | R: ACGCCATGGTTATGTCCTTGA |  |  |
